# Rapeseed Protein–Fiber Concentrate as a Novel Ingredient for Pasta Production: Technological and Quality Characteristics

**DOI:** 10.3390/gels12070560

**Published:** 2026-06-23

**Authors:** Marina Axentii, Georgiana Gabriela Codină, Juan E. Andrade Laborde, Aurelian Rotaru

**Affiliations:** 1 Faculty of Food Engineering, Stefan cel Mare University of Suceava, 720229 Suceava, Romania; axentiimarina@gmail.com; 2 Department of Food Science and Human Nutrition, University of Florida, Gainesville, FL 32611, USA; jandrade2@ufl.edu; 3 Science & Research Center MANSiD, Faculty of Electrical Engineering and Computer Science, Stefan cel Mare University of Suceava, 720229 Suceava, Romania; aurelian.rotaru@usm.ro

**Keywords:** fiber, protein, pasta, rapeseed, sustainability

## Abstract

The aim of this study was to evaluate the possibility of using rapeseed protein–fiber concentrate (RPFC) as a functional ingredient for wheat pasta fortification, with emphasis on dough rheology, gel-like network formation, microstructure, and cooking quality. For this purpose, five formulations of rigatoni pasta were produced by partially substituting wheat flour with 0, 5, 10, 15, and 20% RPFC. Dough rheological behavior was assessed by frequency sweep and creep–recovery tests, while mixing and pasting behavior was evaluated using the Mixolab device. Microstructure was analyzed by scanning electron microscopy (SEM), and pasta technological and chemical parameters were determined using standard methods. All dough systems exhibited viscoelastic, gel-like behavior characterized by the dominance of the storage modulus (G’) over the loss modulus (G”), confirming the formation of a structured gluten-based network. Moderate RPFC incorporation (5–15%) enhanced G′, indicating reinforcement of the continuous protein–starch gel matrix and improved structural integrity and deformation resistance. Mixolab results showed a significant increase in water absorption and dough stability with RPFC addition, reflecting improved hydration and strengthening of the gel-forming protein network. SEM observations confirmed the development of a more compact and continuous starch–protein gel system, associated with reduced stickiness and improved structural cohesion. However, higher RPFC levels (15–20%) disrupted the continuity of the gel network, leading to increased cooking losses (8.8–10.4%), higher fracturability, and reduced firmness of cooked pasta. According to the data obtained, RPFC represents a promising functional protein ingredient for gel-like food systems such as cereal-based products, particularly pasta. These findings offer feasible formulation strategies and support its use as a sustainable, high-quality plant protein ingredient in pasta production.

## 1. Introduction

Given global population growth and, therefore, the increase in food requirements, there is a need for new, sustainable sources that can support a healthy diet. According to projections by the Food and Agriculture Organization (FAO) [1], the global population is expected to approach 9.8 billion by 2050, accompanied by a substantial increase in demand for high-quality dietary protein. In particular, the consumption of animal-source proteins is projected to increase by approximately 60–70% by 2050, due to population growth, urbanization, and changing dietary patterns [1,2]. Meeting this demand exclusively through conventional animal production will eventually lead to environmental destruction as it will result in high greenhouse gas emissions, intensive land and water use, and pressure on natural resources. If we reside mostly on conventional livestock production, the environmental response by 2050 is expected to be largely negative and cumulative. Since food insecurity persists globally, finding new protein sources may be the key to drastically reducing it. Consequently, there is a growing interest in sustainable plant-based protein alternatives, especially those derived from oilseed by-products such as rapeseed, which offer high nutritional value, significant amounts of dietary fibers, protein quality, and bioactive compounds while also aligning with circular-economy principles [1].

The incorporation of rapeseed protein–fiber concentrate (RPFC) obtained from the by-products of rapeseed oil production, specifically from rapeseed press cake, a by-product that mostly goes to waste [2] into staple foods such as pasta represents a promising strategy to enhance protein and fiber intake of the population, contributing to the overall sustainability of food systems.

Besides food insecurity concerns, rapeseed is the second most widely cultivated oilseed crop worldwide [3]; therefore, there is a global supply of rapeseed that can be used in the food industry. Advances in processing technologies have enabled the production of food-grade rapeseed protein isolates and concentrates with reduced antinutritional factors and improved sensory properties, making them increasingly suitable for food applications.

Globally, the safety of rapeseed and canola products is also supported by many organizations. In the EU, rapeseed protein ingredients are regulated under Novel Food Regulation (EU) 2015/2283 (Regulation (EU) 2015/2283 of the European Parliament and of the Council of 25 November 2015 on novel foods 2015) while in the U.S., rapeseed/canola protein ingredients are marketed under the Generally Recognized as Safe (GRAS) framework following UnitedStates Food and Drug Administration (FDA) [4], meaning that food-grade rapeseed protein ingredients are safe when manufactured and used according to defined protocols. As a food ingredient, rapeseed protein exhibits favorable technological functionalities, including water absorption, emulsification, foaming, and gel-forming capacity [1].

These properties allow its incorporation into a wide range of food products, including bakery goods, meat analogs, dairy alternatives, and protein-enriched pasta, where it can improve protein content while maintaining acceptable texture and cooking quality [5,6,7,8].

In pasta products, the inclusion of protein–fiber concentrates such as Raptein®30, a novel food ingredient developed by Napiferyn Biotech, is of particular interest due to its potential impact on the formation and stability of the gluten-based protein gel network, which directly affects dough viscoelasticity [9,10]. In wheat-based pasta dough, the gluten matrix can be described as a weak viscoelastic gel formed through hydration-induced interactions between glutenin and gliadin proteins [11]. This gel-like network is responsible for the mechanical integrity of the dough and plays a central role in determining processing behavior and final product quality.

The incorporation of Raptein®30 introduces additional compounds, particularly proteins and dietary fiber components, that may modify gel formation by altering water distribution, competing for hydration, and interacting with gluten proteins [10]. The fiber fraction may disrupt or spatially reorganize the protein gel network, whereas the protein fraction may contribute to network reinforcement or partial co-gelation phenomena, depending on processing conditions [9,12]. These modifications are particularly important, as they directly influence dough handling properties, processing performance, and the structural integrity of the final pasta product.

Morphologically, rapeseed fibers appear similar to wood fibers, being mainly composed of cellulose (48.5%) [13], while also containing lignin, holocellulose, pentosans, and minor amounts of ash [14,15]. Additionally, rapeseed proteins provide a relatively balanced essential amino acid profile, with appreciable levels of sulfur-containing amino acids (methionine and cysteine), as well as lysine and threonine, which are often limiting in wheat proteins [16]. These characteristics may improve both the biological value of proteins and the dietary fiber content of pasta products.

Therefore, the present study aimed to develop pasta enriched with both high-quality plant protein and naturally occurring dietary fiber while preserving desirable technological and quality characteristics through the incorporation of Raptein®30, an innovative food-grade ingredient for pasta production. This work follows our previous study [17], which demonstrated the potential of Raptein®90 to improve the nutritional and technological quality of pasta products.

## 2. Results and Discussion

### 2.1. Characterization of RPFC-Enriched Pasta Formulations

**Figure 1:**
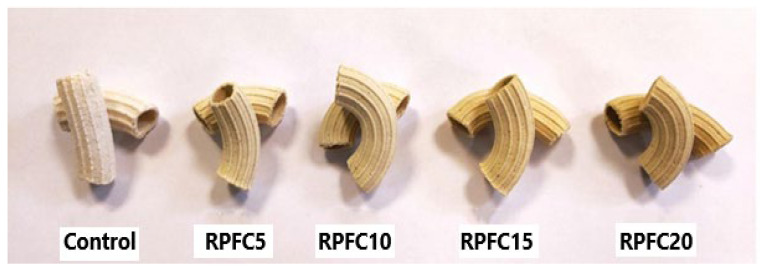
Rigatoni pasta enriched with various levels of rapeseed protein–fiber concentrate (RPFC): Control—0%; RPFC5—5%; RPFC10—10%; RPFC15—15%; RPFC20—20%.

Figure 1 shows RPFC-enriched pasta samples after drying, arranged from left to right as per increasing substitution levels of wheat flour with RPFC.

As may be seen, dried rigatoni pasta appears to have a compact and well-defined profile, the ridged external surface remains uniform among all pasta samples, and the tubular geometry is maintained without collapse, cracking, or excessive warping. Compared to the control sample, an improvement of the pasta texture and extrudability was observed. Also, the addition of protein–fiber rapeseed concentrate contributed to an intensification of pasta color, ranging from a light off-white shade to a brown–olive color characteristic of RPFC20 containing 20% RPFC. The color changes are consistent with findings reported in similar studies. Sadeghi and Bhagya [18] reported that spaghetti fortified with 2.5–10% mustard protein isolate exhibited increased color saturation (higher a* and b* values) and a significant reduction in lightness as the substitution level increased. Furthermore, browning can be attributed to Maillard reactions occurring during the drying process, a trend also reported in pasta formulations containing 15–20% hemp flour [19]. This phenomenon is also associated with the presence of free amino acids and residual sugars that are sensitive to elevated temperatures.

On top of that, dried rigatoni pasta appears to have a compact and well-defined profile; the ridged external surface remains uniform among all pasta samples, and the tubular geometry is maintained without collapse, cracking, or excessive warping. Compared to the control sample, an improvement of the pasta texture and extrudability was observed.

### 2.2. Dough Rheological Characteristics

**Figure 2:**
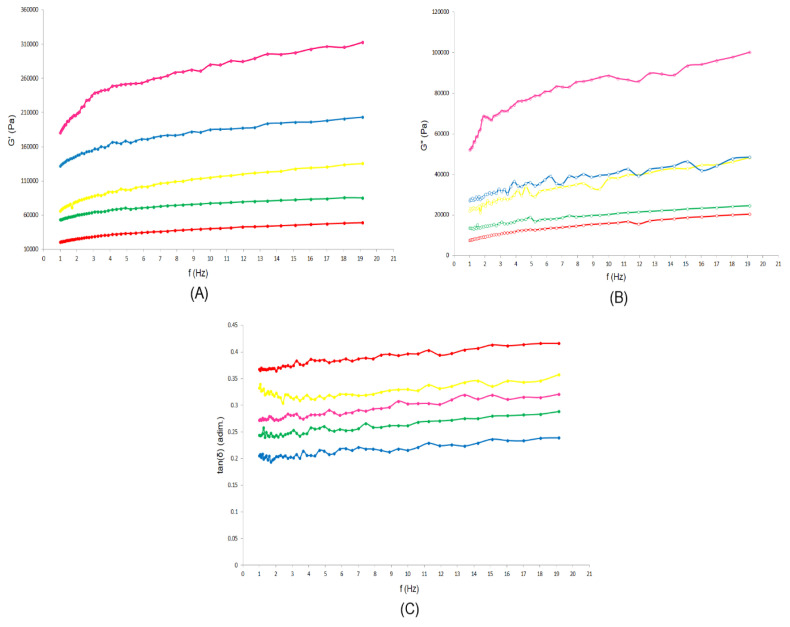
Dynamic frequency sweep analysis of pasta dough enriched with various levels of rapeseed protein–fiber concentrate (RPFC): (A) – storage modulus (G’, solid symbols); (B) – loss modulus (G”, empty symbols); (C) – loss tangent (tan δ, solid symbols); -•- control; -•- RPFC5 (5%); -•- RPFC10 (10%); -•- RPFC15 (15%); -•- RPFC20 (20%).

For all dough samples, frequency sweep analysis showed an increase in the storage modulus (G′), indicating predominantly elastic and gel-like behavior, as may be seen in Figure 2. This response can be attributed to enhanced protein–water–starch interactions, which favor the formation of a more continuous gel network and increase the rigidity of the dough matrix [20,21]. Similar gel-strengthening effects have been reported in dough systems enriched with fiber-rich ingredients such as grape seed flour, psyllium, and soluble barley fibers, where increases in G′ were associated with the development of stronger viscoelastic gel structures [22,23,24]. In particular, RPFC20, containing the highest RPFC substitution level (20%), exhibited the second-highest G′ values and the lowest tan δ values, suggesting the formation of a highly elastic and rigid dough structure. This behavior may be attributed to the combined contribution of rapeseed proteins and dietary fibers, which enhance water immobilization and increase the concentration of solid components within the dough system. Rapeseed storage proteins, mainly cruciferin (11S globulin) and napin (2S albumin), interact with gluten proteins through non-covalent interactions, including hydrogen bonding and hydrophobic interactions, promoting additional physical crosslinks within the gluten–starch matrix [25]. Furthermore, polysaccharides present in the RPFC fiber fraction contain numerous hydroxyl (–OH) groups that facilitate water-binding and hydrogen bond formation [26,27]. This enhanced water immobilization reduces molecular mobility and increases matrix density, contributing to higher G′ values and more pronounced gel-like behavior (G′ > G″). As shown in Figure 2C, loss tangent (tan δ) values remained less than 1 for all formulations, confirming the formation of a solid-like viscoelastic gel system [19]. Since tan δ represents the ratio of loss modulus (G″) to storage modulus (G′), values lower than 1 (tan δ < 1) indicate a predominance of elastic (gel-like) behavior over viscous flow [28,29]. Lower tan δ values are typically associated with stronger, more stable gel networks and improved structural integrity, whereas increases in tan δ reflect progressive weakening of the gel matrix [29,30,31]. Dough samples with a high degree of cross-linking are expected to have a low tan δ [32]. Accordingly, the lower tan δ values observed in RPFC-enriched formulations suggest the formation of a more stable and cohesive gel-like structure, which is favorable for pasta processing.

**Figure 3:**
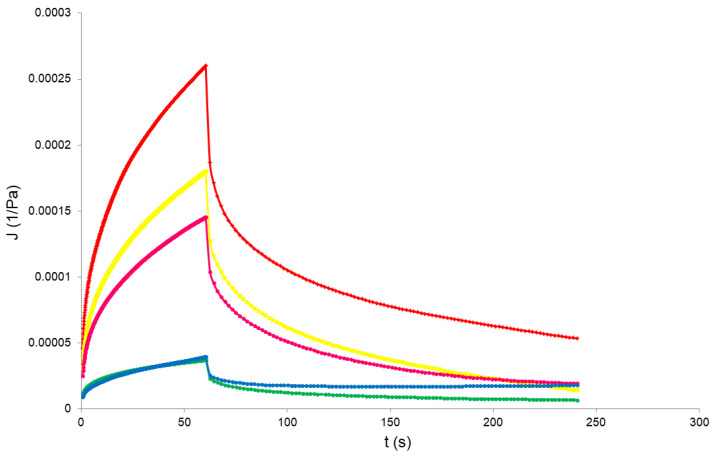
Creep–recovery response of pasta dough enriched with various levels of rapeseed protein–fiber concentrate (RPFC): -•- control; -•- RPFC5 (5%); -•- RPFC10 (10%); -•- RPFC15 (15%); -•- RPFC20 (20%).

All dough samples exhibited typical viscoelastic creep–recovery behavior, characterized by increasing strain under applied stress, followed by partial recovery upon stress removal, as it may be seen in Figure 3. Higher RPFC levels (15–20%) resulted in decreased compliance and increased recovery, indicating the formation of a more elastic dough matrix with higher resistance to stress. Table 1, which summarizes the creep and recovery parameters, shows a decrease with increasing RPFC incorporation in instantaneous elastic compliance (Je) from 4.77 × 10^−5^ Pa^−1^ to 1.96 × 10^−5^ Pa^−1^ and maximum creep compliance (J_max_), from 8.26 × 10^−5^ Pa^−1^ to 3.94 × 10^−5^ Pa^−1^ respectively, suggesting reduced deformability and the development of a more rigid and compact gel matrix. Simultaneously, the viscosity coefficient (η) significantly increased from the control to the RPFC20 sample, indicating enhanced resistance to viscous flow and a higher degree of structural consolidation within the protein–starch gel network. Overall, these results suggest that RPFC incorporation increased dough rigidity and resistance to deformation, mainly due to the contribution of rapeseed proteins and fibers within the dough matrix. However, the replacement of wheat flour with greater amounts of RPFC reduces gluten-forming proteins, which may limit the development of a continuous gluten network and result in a more rigid but less homogeneous (more porous) dough structure [32].

### 2.3. Dough Rheological Properties During Mixing and Pasting

The Mixolab data (Table 2) highlighted the impact of rapeseed protein–fiber concentrate (RPFC) addition on dough behavior during mixing and heating.

As may be seen, water absorption (WA) significantly increased from 55% (control) to 90% (RPFC20). The increase in water absorption can be attributed to the high water-binding capacity of RPFC, due to the presence of polar functional groups in its structure. Similar trends have been reported for dough systems enriched with other plant proteins, including hemp, pea, and soy proteins [33,34,35] or fibers [36,37]. Dough stability showed a slight improvement with increasing RPFC addition, reaching values above 12 min for the RPFC10 and RPFC20 samples. This behavior indicates enhanced resistance of the dough gel network to mechanical and thermal stress, which is particularly relevant for extrusion-based pasta processing [38]. The C2 torque, which indicates the protein weakening during heating, increased for the RPFC-enriched samples, indicating a more compact protein network. However, at 20% RPFC addition, C2 begins to slightly decrease, probably due to the gluten dilution effect. These results indicate that moderate levels of RPFC may reinforce the protein network, whereas excessive substitution may limit this effect [39,40]. Regarding starch gelatinization behavior, a significant decrease in C3 torque values was observed at higher RPFC levels, mainly due to starch dilution and competition for water between starch granules, fiber, and protein fractions from RPFC. The starch gelatinization rate, expressed by the β slope, remained relatively constant across most formulations but decreased significantly for the RPFC20 sample (0.358 Nm/min), indicating a slower or less efficient gelatinization process. This behavior is consistent with previous studies on wheat–hemp flour systems, where protein–starch interactions influenced gel formation at elevated temperatures [41]. Furthermore, the values for hot starch paste torque (C4) significantly decreased with increasing RPFC content, suggesting the formation of a less rigid gel structure. This effect may have positive effects for pasta storage quality, as reduced retrogradation (C5) is associated with improved textural stability over time. Similar reductions in C5 values have been reported for bread formulations enriched with soy protein and dietary fibers [42].

### 2.4. Dry Pasta Microstructure Evaluation

The microstructure of pasta is an important qualitative indicator of the texture for the final product, especially for identifying micro-cracks or technological defects caused by the drying process that are not visible to the naked eye.

**Figure 4:**
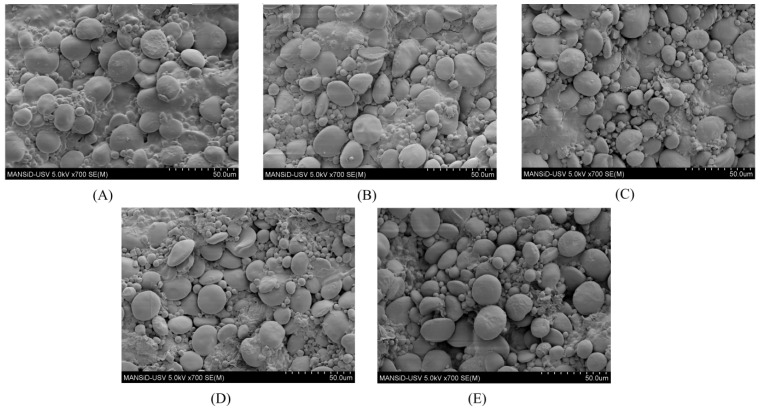
SEM evaluation of pasta enriched with various levels of RPFC: (**A**)—control (0%); (**B**)—RPFC5 (5%); (**C**)—RPFC10 (10%); (**D**)—RPFC15 (15%); (**E**)—RPFC20 (20%).

According to the SEM micrograph of the control sample (Figure 4A), the pasta has a compact and well-developed matrix consisting of starch granules embedded within a continuous gluten network. The starch granules appear largely intact and well incorporated into the protein phase, with very few voids or discontinuities. The surface is relatively smooth, indicating well-established starch–gluten interactions. This type of morphology is characteristic of durum wheat doughs with a coherent gluten network, and it is commonly associated with good structural integrity of pasta products.

For both samples containing 5% RPFC (Figure 4B) and 10% RPFC (Figure 4C), the structure remains generally compact; however, slight discontinuities in the protein network can be observed. Some starch granules are partially exposed, and small localized aggregates appear in certain regions. However, at 15% RPFC enrichment (Figure 4D), the structure appears to be porous and heterogeneous. The presence of RPFC, rich in both proteins and dietary fiber, interferes with the formation of a uniform protein network. In addition, increased surface roughness is observed, suggesting reduced dough cohesion and weaker structural continuity. In the sample containing 20% RPFC (Figure 4E), starch granules are irregularly aggregated, and the protein matrix is highly fragmented and insufficient to uniformly entrap the starch phase. Pronounced voids are evident between granules, resulting in a highly porous and disorganized structure.

### 2.5. Fracturability Analysis

The increase in pasta fracturability with RPFC addition indicates the formation of a less cohesive and more brittle structure in the dried pasta samples. During dough mixing and extrusion, RPFC promoted the development of a more elastic and structured dough, as shown by the higher G′ values obtained during rheological analysis. However, after drying, the pasta structure became more rigid and easier to break.

This behavior may be explained by the combined effect of rapeseed proteins and fibers on the gluten–starch matrix [43]. The improved viscoelastic properties observed in the pasta dough hydrated state do not necessarily translate into higher mechanical resistance after drying. Moreover, the presence of fiber-rich particles promotes the formation of pores and structural discontinuities within the dried pasta matrix. These pores and structural discontinuities act as weak points that facilitate crack formation and propagation during mechanical stress, resulting in increased brittleness despite the higher elasticity observed in the dough stage [24]. In addition, the high water-binding capacity of RPFC may have influenced moisture distribution during drying, further contributing to the rigidity of the final pasta texture, similar to findings reported by Itusaca-Maldonado et al. [44] in the case of quinoa protein-enriched pasta dough. As a result, although RPFC improved dough elasticity during processing, the dried pasta samples exhibited higher fracturability, especially at higher substitution levels.

**Figure 5:**
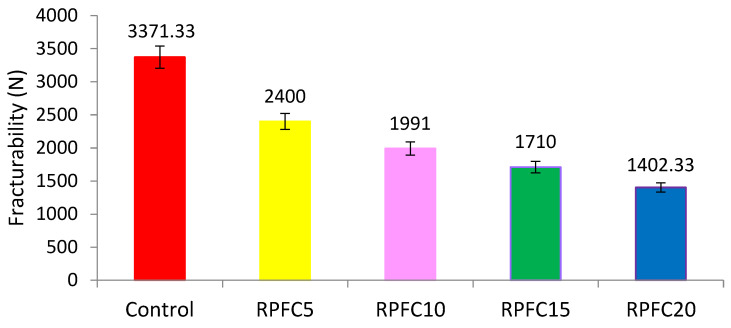
Fracturability test performed on dry pasta samples (control—0%RPFC; RPFC5—5% RPFC; RPFC10—10% RPFC; RPFC15—15% RPFC; RPFC20—20% RPFC).

As shown in Figure 4, increased surface roughness, enhanced brittleness in the RPFC15 and RPFC20 samples, as well as irregular edges, were further supported by the fracturability test performed on dry pasta samples (Figure 5).

The increased fracturability of RPFC15 and RPFC20 samples can be primarily attributed to the dilution of the gluten network resulting from the progressive substitution of wheat flour with RPFC. Numerous studies on pasta enriched with rapeseed, pea, lentil, chickpea, and soy proteins have reported that a substitution range of 5–15% is generally optimal for obtaining products with a compact structure while providing nutritional benefits [45]. Exceeding an approximate threshold of 15% added protein tends to induce evident adverse effects, including excessive hardness or undesirably increased fracturability.

### 2.6. Technological Properties

The evaluation of technological parameters, including optimal cooking time (OCT), water absorption (WA), cooking loss (CL), and swelling index (SI) (Table 3), represents a key element in the assessment of pasta cooking quality, as these four parameters directly influence the sensory profile of the final product.

A decrease in optimal cooking time was observed, from 14 to 10 min. At the same time, protein-enriched pasta tended to absorb larger amounts of water, exhibiting a less firm texture compared to the control sample. A decrease in OCT values may be due to RPFC water-binding capacity [8,46]. A similar trend was reported in pasta enriched with legume flours and dietary fibers [34,47]. Under normal conditions, gluten in wheat forms an elastic three-dimensional network surrounding starch granules, thereby slowing water diffusion and starch gelatinization. According to different researchers [8,10], when part of the gluten is replaced by rapeseed proteins, which are unable to form a gluten network and dietary fiber, the matrix becomes discontinuous and less dense, allowing water to penetrate the pasta structure more easily. Consequently, starch gelatinization occurs earlier, leading to faster elimination of the “white core,” as also reflected by the increase in swelling index (SI) values (from 1.91 to 2.31). However, there are studies reporting an increase in cooking time following protein addition. For instance, in pasta enriched with 10% defatted soy flour, the structure became more compact, as soy protein–fiber can act as an emulsifier, leading to increased sample density and, consequently, longer cooking times [48]. In the case of pasta enriched with rapeseed protein–fiber concentrate, its composition (approximately 60–70% protein and 20–30% fiber) is comparable to that of hemp seed meal.

The increase in cooking loss induced by RPFC addition can be attributed to structural modifications of the gluten–starch matrix resulting from the combined effects of the protein and fiber fractions. The integrity of the gluten network relies on strong covalent disulfide (S–S) bonds and numerous cumulative weak interactions between extensible polypeptide chains. In contrast, rapeseed proteins are globular in nature, with folded structures stabilized internally, and therefore cannot establish the same extensive interactions with gluten. Their interactions are limited to hydrogen bonding or weak hydrophobic forces at the surface, potentially leading to the formation of separate protein–protein complexes (rapeseed protein aggregates) during heating, which do not contribute to the mechanical strength of the starch-holding network [48,49]. As starch granules begin to hydrate and swell at approximately 60–70 °C, their osmotic pressure is no longer counterbalanced by an intact gluten “cage,” leading to granule rupture and amylose leaching into the surrounding medium. Being a linear polymer, amylose diffuses into the cooking water, increasing its viscosity and adhering to the pasta surface, thereby promoting stickiness [48]. Additionally, RPFC fiber fraction mainly consists of insoluble compounds, such as cellulose and lignin, together with soluble components, including hemicellulose, pectins, gums, β-glucans, and mucilage [9,50]. Insoluble fibers can act as physical barriers within the gluten network, disrupting matrix continuity, while soluble fibers compete for water availability due to their high hydration capacity. Therefore, as the RPFC level increases from 5% to 20%, cooking losses tend to rise due to the progressive weakening of the gluten network, increased fiber content, and the increased exposure of starch to water.

### 2.7. Total Phenolic Content (TPC), Total Flavonoid Content (TFC), and Antioxidant Capacity (DPPH) of RPFC-Enriched Pasta

Table 4 presents the variations in DPPH antioxidant activity (expressed as %), total phenolic content (TPC), and total flavonoid content (TFC) of the samples obtained by partial substitution of wheat flour with RPFC (5–20%) addition. The results indicate a complex relationship between RPFC concentration and the extracted bioactive compounds.

The antioxidant activity (DPPH) increased significantly in all RPFC-enriched samples compared with the control sample (1.59%), reaching a maximum of 2.67% Inhibition of DPPH in the RPC10 pasta sample. This enhancement in antioxidant activity can be attributed to the presence of rapeseed-specific phenolic compounds, such as sinapic acid, ferulic acid, and their derivatives, which are well known for their free-radical scavenging capacity [34]. An interesting result is that DPPH values remained relatively high in the RPC15 and RPC20 samples (2.38–2.51%), despite a significant decrease in TPC, suggesting a potentially higher contribution from flavonoids or other non-phenolic antioxidant compounds (e.g., bioactive peptides or sulfur-containing compounds) at higher RPFC levels.

Total phenolic content (TPC) was highest in the control sample (358.29 mg GAE/kg) and decreased progressively with increasing RPFC addition, reaching 125.47 mg GAE/kg in RPC20. This apparently contradictory trend may be explained by the dilution of the naturally occurring polyphenols present in wheat flour, as well as by the possible degradation of heat-sensitive phenolic compounds during drying or extrusion processes. In addition, certain RPFC components may interfere with colorimetric quantification methods (Folin–Ciocalteu), potentially leading to an underestimation of the actual phenolic content [51]. On top of that, certain rapeseed-derived phenolic compounds, particularly redox-active phenolics, may interact with gluten proteins, influencing thiol/disulfide exchange reactions and modifying gluten network development. During dough formation and thermal processing, these compounds may interact with amino acid side chains or become incorporated into protein complexes, reducing their extractability and consequently contributing to the lower TPC values detected [52]. Therefore, the observed reduction in TPC may not necessarily indicate a loss of phenolic compounds but rather their possible involvement in protein–phenolic interactions within the RPFC-enriched pasta matrix.

In contrast, total flavonoid content (TFC) showed a non-linear trend. The highest value was observed in the control sample (227.46 mg QE/kg), while RPFC addition generally reduced TFC, with the lowest value recorded for RPFC10 (59.93 mg QE/kg). However, TFC increased again at higher RPFC levels, reaching 150.99 mg QE/kg for RPFC15 and 164.71 mg QE/kg for RPFC20. The non-linear trend can be attributed to several reasons: inadequate dilution factor, reduced extractability of flavonoid compounds due to protein–polyphenol interactions, and possible matrix-related interference during colorimetric determination. Moreover, at moderate substitution levels (RPFC5–RPFC10), these compounds may be less efficiently solubilized or may form complexes with proteins, thereby reducing their extractability and subsequent qualitative and/or quantitative detection. The higher TFC observed in RPC20 indicates a more effective release of flavonoids from the protein matrix, likely due to the more porous dough structure at elevated RPFC levels.

### 2.8. In Vitro Protein Digestibility and PDCAAS Score Evaluation

The incorporation of rapeseed protein–fiber concentrate (RPFC) into wheat-based pasta resulted in a significant improvement in protein quality, as evidenced by the progressive increase in PDCAAS values (from 56 to 99%), while in vitro digestibility remained relatively constant across all formulations (Table 5). This trend is consistent with previous studies on protein-enriched cereal products, where digestibility is often only marginally affected, whereas protein quality improves due to amino acid complementation. Wheat-based products such as conventional pasta are inherently deficient in lysine, since the main proteins are gliadins and glutenins, with lysine being a limiting amino acid, which restricts their biological value. In contrast, rapeseed proteins are characterized by a favorable amino acid profile, being particularly rich in lysine and sulfur-containing amino acids [53]. Consequently, the addition of RPFC becomes complementary, increasing the lysine content of wheat flour (Table 6), thereby correcting the amino acid imbalance and enhancing the overall protein quality of the final product. Similar improvements have been reported in studies evaluating rapeseed protein isolates and other plant-based enrichments, where PDCAAS values approached those of high-quality proteins despite minimal changes in digestibility [54]. Furthermore, it is well established that PDCAAS is primarily influenced by the limiting amino acid rather than digestibility alone, explaining the marked increase in protein quality observed in RPFC-enriched samples. Present findings confirm that RPFC acts as an effective complementary protein source in cereal-based systems, fortifying pasta by improving nutritional quality while preserving high protein digestibility.

### 2.9. Chemical Profile

Table 7 highlights the impact of rapeseed protein–fiber concentrate (RPFC) addition on the chemical composition of pasta samples. Progressive incorporation of RPFC (5–20%) resulted in a significant increase in protein content, from 10.40% in the control sample to 17.11% in RPFC20, confirming the high nutritional value of rapeseed protein [34]. According to current EU legislation, RPFC10, RPFC15, and RPFC20 can be considered “source of protein” since at least 12% of their energy value is given by protein [55]. The increase in ash content (from 0.60% to 1.54%) indicates an enhanced mineral contribution, characteristic of oilseed meals [35,48,49]. In addition, the moisture content slightly decreased with increasing RPFC levels, from 11.8% to 10.40% in RPFC20.

### 2.10. Corrected ATR–FTIR Analysis

**Figure 6:**
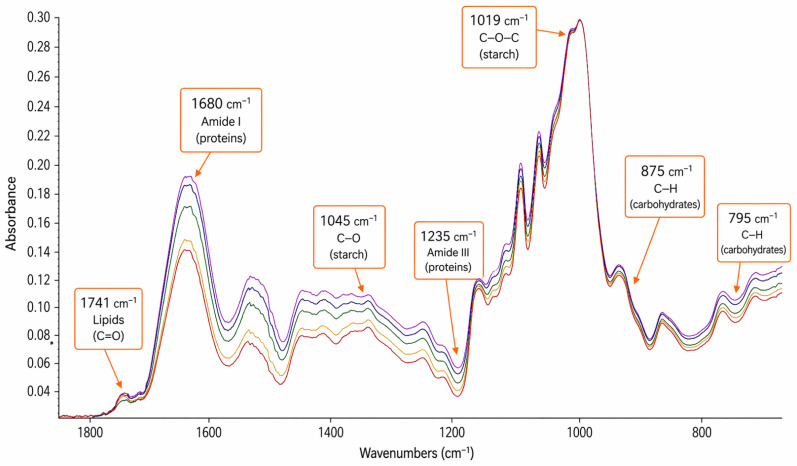
ATR–FTIR spectra of protein-enriched pasta samples with varying RPFC substitution levels: -- control; -- RPFC5 (5%); -- RPFC10 (10%); -- RPFC15 (15%); -- RPFC20 (20%).

The ATR–FTIR spectra of pasta enriched with rapeseed protein–fiber concentrate (RPFC), shown in Figure 6, highlight structural modifications of the pasta matrix and allow the identification of specific chemical groups associated with RPFC, particularly in the RPFC15 and RPFC20 samples.

The absorption band around 1741 cm^−1^ is associated with C=O stretching vibrations of esters or fatty acids, indicating the presence of lipid components or traces of compounds derived from plant proteins [56,57].

The band at approximately 1680 cm^−1^ corresponds to amide I vibrations, characteristic of C=O stretching present in various plant-based protein sources, including but not limited to rapeseed protein structures, while the region around 1540–1550 cm^−1^ (amide II) is related to N–H bending and C–N stretching vibrations, suggesting the presence of peptide bonds [58].

Additionally, the bands observed at 1235 cm^−1^ and 1045 cm^−1^ are attributed to P=O, C–O, and C–OH stretching vibrations, which are characteristic of polysaccharides and native or modified starch. The pronounced peak at 1019 cm^−1^ can be associated with C–C and C–O–C vibrations present in carbohydrate networks. The bands at 875 cm^−1^ and 795 cm^−1^ correspond to CH wagging vibrations, typically associated with cyclic structures or aromatic components, which may originate from dietary fiber or residual fractions of RPC [59].

Overall, ATR–FTIR spectra indicated changes in the molecular structure of RPFC-enriched pasta samples, suggesting the incorporation of rapeseed-derived components and possible interactions within the protein–starch matrix. However, considering the complexity of food systems and the overlapping nature of FTIR absorption bands, these results should only be taken into consideration as supportive evidence of possible molecular interactions and structural modifications associated with RPFC addition.

### 2.11. Sensory Profile Evaluation

**Figure 7:**
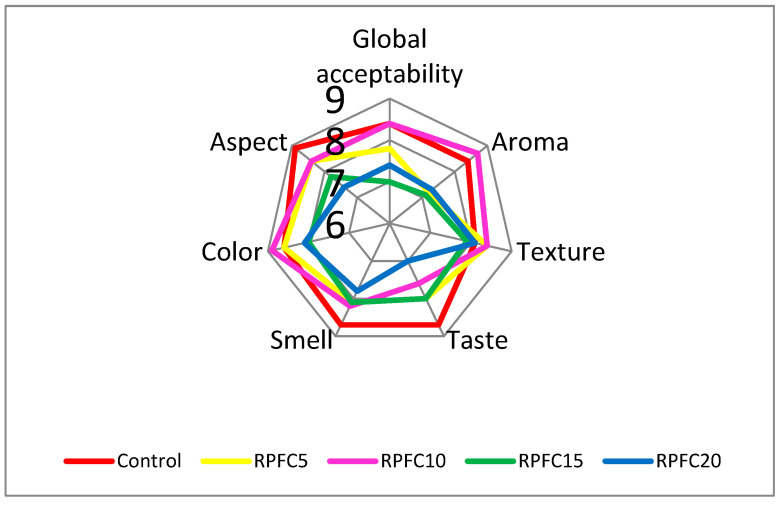
Sensory profile evaluation of pasta enriched with RPFC.

Figure 7 highlights the sensory profile evaluation of pasta samples enriched with RPFC using a 9-point hedonic scale. As expected, the control sample received the highest sensory scores, particularly for aspect, smell, and taste, reflecting the panelists’ familiarity and interest in conventional wheat-based pasta, a staple product of Italian cuisine.

Among the enriched samples, RPFC10 recorded the highest sensory performance, with a global acceptability score of 8.4 out of 9. The lowest score within this formulation was observed for taste (7.6), as panelists reported an earthy and slightly bitter flavor characteristic for rapeseed, most likely due to the presence of phenolic compounds and other bioactive constituents naturally occurring in RPFC. Higher substitution levels in RPFC15 and RPFC20 led to a decrease in global sensory acceptability, as rapeseed flavor intensified and higher substitution levels slightly altered the texture (softer pasta) and mouthfeel. This reduction may be attributed to the intensified rapeseed flavor, increased fiber content, and structural modifications of the gluten network. Despite these changes, all fortified samples maintained sensory scores above 7, demonstrating the potential applicability of RPFC as a functional ingredient in pasta formulations.

## 3. Conclusions

The results of this study demonstrate that RPFC can be effectively used as a functional ingredient in wheat-based pasta, enhancing protein content while improving dough elasticity and providing satisfactory cooking performance. The observed effects—ranging from gluten network dilution and increased water absorption to modifications in texture, digestibility, and antioxidant activity—highlight the complex and non-linear behavior of RPFC within the pasta matrix.

From a structural perspective, the present study provides valuable insights regarding the industrial feasibility of RPFC-enriched pasta, as moderate incorporation levels were able to resist thermal processing and mechanical stress, both of which represent critical control points during industrial pasta manufacturing. In particular, formulations containing 5 and 10% RPFC showed the best balance between improved rheological behavior, structural stability, cooking quality, and sensory acceptability. Rheological analysis confirmed the formation of predominantly elastic and gel-like dough systems, while microstructural observations confirmed a continuous and compact protein–starch matrix.

Nutritionally, RPFC enrichment led to increased protein content, fiber content, and significant amounts of bioactive compounds in pasta formulations. The presence of rapeseed-derived phenolic compounds may further enhance the functional potential of these products through improved antioxidant properties. Therefore, RPFC represents a promising multifunctional ingredient capable of combining technological functionality with nutritional enhancement in cereal-based foods like rigatoni pasta.

From a broader perspective, this work contributes to the valorization of rapeseed processing co-products within a circular-economy framework by demonstrating their applicability in human nutrition rather than conventional low-value feed applications. Moreover, the present findings provide valuable insight into the interactions between rapeseed proteins, starch, and gluten, which may help researchers and food manufacturers better understand the behavior of rapeseed protein concentrates in pasta systems and support their rational application in the development of fortified and sustainable food products.

## 4. Materials and Methods

### 4.1. Materials

The main raw materials used for the formulation of protein-enriched pasta were wheat flour type 650 (S.C. Mopan, Suceava, Romania) and rapeseed protein–fiber concentrate (RPFC, commercial name Raptein^®^30; Napiferyn Biotech Sp. z o.o., Łódź, Poland). The wheat flour presented the following data, determined according to international standard methods: 14.2% moisture content (ICC 110/1), 11.6% protein content (ICC 105/2), 0.65% ash content (ICC 104/1), 1.12% fat content (ICC 136), 378 s falling number (ICC 107/1), and 29.8% wet gluten content (ISO 21,415–2:2015). According to the manufacturer, the rapeseed protein–fiber concentrate (RPFC, Raptein®30) batch used in this study was characterized by the following compositional profile: moisture content of 1.6%, protein content of 35.3%, fat content of 0.4%, ash content of 3.0%, carbohydrates of 61.4%, and total dietary fiber content of 60.9%. In addition, RPFC was developed by NapiFeryn BioTech (Łódź, Poland) through a patented technology that allows the production of food-grade proteins from rapeseed. According to the manufacturing company, the biomass remaining after oil extraction was processed through a safe, innovative, and efficient technology to isolate nearly all protein from rapeseed without using harmful chemical compounds such as hexane. According to the European Commission, under Regulation (EU) 2015/2283, Raptein®30 has been authorized as a food ingredient, being a natural mix of protein (24–45%) and dietary fiber (35–70%) with high oil and water absorption capacity [60,61].

### 4.2. Methods

#### 4.2.1. Pasta Formulation

Protein-enriched rigatoni pasta samples containing various levels of protein–fiber concentrate (RPFC) were obtained as follows: control (0% RPFC), RPFC5 (5% RPFC), RPFC10 (10% RPFC), RPFC15 (15% RPFC), and RPFC20 (20% RPFC). The pasta dough was prepared by first mixing all dry ingredients, namely wheat flour, RPFC, and iodized salt, using a KitchenAid mixer (Whirlpool Corporation, Benton Harbor, MI, USA), followed by the gradual incorporation of water, between 55 and 65% (Table 8), until a homogenous dough was formed. The dough was then extruded using a KitchenAid gourmet pasta press equipped with a rigatoni mold, yielding pasta with an approximate diameter of 16 mm and a length of 50 mm. Drying was carried out in a ventilated oven at a constant temperature of 40 °C for 16 h, followed by cooling to room temperature and storage in airtight ziplock bags.

The hydration capacity of each pasta sample was established based on preliminary trials supported by our previous study performed on rapeseed protein isolate (RPI) enriched pasta formulations [17]. Due to the high protein and dietary fiber content of RPFC and its high water-binding capacity [10], water addition was gradually adjusted to obtain a homogeneous dough, suitable for further extrusion. In addition, according to existing FDA regulations [62], dried pasta products should contain at least 87% total solids, corresponding to a maximum moisture content of 13%.

#### 4.2.2. Rheological Properties of Dough and Pasta

##### Dough Dynamic Rheology

Dynamic rheological properties of dough were evaluated using a Thermo-HAAKE MARS 40 rheometer (Thermo Fisher Scientific, Karlsruhe, Germany), following the method described by Axentii et al. [19]. A frequency sweep test was conducted on dough samples, previously rested for 30 min at room temperature to eliminate internal stress. The following parameters were measured: storage modulus (G′), loss modulus (G″), and loss tangent (tan δ). A parallel-plate geometry (40 mm diameter, 2 mm gap) was used, with frequency ranging from 0.1 to 20 Hz under a constant stress of 15 Pa. A creep–recovery test was also performed to evaluate the non-linear viscoelastic properties of pasta dough to a time of 60 s and 120 s, respectively, following the Burger model, as described by Hernandez-Estrada [63]. The following parameters were analyzed: Je—elastic compliance; Jv –viscous compliance (creep rate); Jmax—total compliance at end of creep; l-retardation time; η-viscosity; Jr—recovered compliance; Jnr—non-recovered compliance; Jr/Jmax—recovery ratio; and Jr/Jmax—permanent strain ratio.

##### Dough Rheological Properties During Mixing and Pasting

The rheological behavior of dough during mixing and heating was analyzed using a Mixolab device (KPM, Tripette et Renaud, Paris, France) according to the ICC No. 173 standard method. Parameters obtained were water absorption (WA, %), dough development time (DDT, min), dough stability (ST, min), and torques corresponding to protein weakening (C2), starch gelatinization (C3), hot starch stability (C4), and starch retrogradation (C5).

#### 4.2.3. Technological Properties of Pasta

Technological quality was determined according to AACC International Method 66–50, suitable for rigatoni-type pasta with uniform cross-sections. The evaluated parameters included optimal cooking time (OCT), water absorption index (WA), cooking loss (CL), and swelling index (SI), and were previously described by Axentii et al. [17]. For determining the optimal cooking time, ten grams of pasta were boiled in 250 mL of distilled water in a 500 mL beaker at 100 °C. At 30 s intervals, pasta samples were pressed between two glass plates to check for the presence of residual starch granules. OCT was recorded when no visible starch core remained. Water absorption index was calculated using Equation (1):

WA = w_1_ − w_2_/w_2_ × 100 (1)

where w_1_ is the weight of cooked pasta, and w_2_ is the dry weight of uncooked pasta.

For determining cooking loss (CL), cooking water from the WA test was evaporated at 105 °C, and the dry residue was weighed. CL was expressed as g/100 g dry pasta. For the swelling index (SI), cooked pasta was dried at 105 °C for 16 h and weighed. SI was calculated according to Equation (2):

SI = m_1_ − m_2_/m_2_ (2)

where m_1_ is the weight of cooked pasta, and m_2_ is the weight of pasta after drying.

#### 4.2.4. Microstructural Analysis

Surface micrographs of dried pasta were captured using a scanning electron microscope (SEM, Hitachi SU-70, Tokyo, Japan) equipped with an Everhart–Thornley secondary electron detector. Samples were sputter-coated with a ~1 nm gold layer to increase conductivity and minimize charge accumulation. Images were taken at an accelerating voltage of 5 kV and a magnification of 750×.

#### 4.2.5. Fracturability Test

Fracturability of dried pasta was determined using a TVT-6700 texture analyzer (Perten Instruments, Hägersten, Sweden) equipped with an aluminum support (13 mm opening). A trigger force of 50 g and a test speed of 3 mm/s were applied. The maximum breaking force (N) required to fracture the sample into multiple pieces was recorded.

#### 4.2.6. Determination of Antioxidant Activity (DPPH), Total Polyphenolic Content (TPC), and Total Flavonoid Content (TFC) of RPFC-Enriched Pasta

Antioxidant activity (DPPH), total polyphenol content (TPC), and total flavonoid content (TFC) were determined according to standard analytical methods, with few modifications. Extracts were prepared according to the method described by Chetrariu et al. [63], as follows: 2 g of ground sample was mixed with 20 mL of 80% methanol, sonicated for 40 min, followed by 10 min shaking and filtration. Extracts were stored at −20 °C in sealed vials until further analysis.

##### Antioxidant Capacity (DPPH)

Antioxidant capacity was determined by measuring absorbance at 517 nm using a UV–VIS–NIR spectrophotometer (Shimadzu Corporation, Kyoto, Japan), according to the methods described by Codină [28] and Chetrariu et al. [64]. A 0.2 mL aliquot of diluted extract (1:5, *v*/*v*) was mixed with 2 mL of 0.1 mM DPPH solution, shaken for 2 min, and incubated in the dark for 30 min at room temperature. Measurements were performed in triplicate, using DPPH solution as a blank. Results were calculated according to Equation (3).

DPPH inhibition% = (1 − A_1_/A_0_) × 100 (3)

where A_0_ is the absorbance of the blank, and A_1_ is the absorbance of the sample.

##### Total Polyphenol Content (TPC)

Total phenolic content (TPC) was determined using the Folin–Ciocalteu method, following the procedure described by Codină et al. [29]. Briefly, 0.2 mL of extract was mixed with 2 mL of Folin–Ciocalteu reagent (previously diluted 1:10, *v*/*v*) and 1.8 mL of sodium carbonate solution (7.5%, *w*/*v*). The reaction mixture was incubated in the dark for 30 min at room temperature, after which the absorbance was measured at 750 nm using a spectrophotometer. A calibration curve was made using gallic acid (10–200 mg/L). Results were expressed as milligrams of gallic acid equivalents per kilogram of dry pasta (mg GAE/kg).

##### Total Flavonoid Content (TFC)

TFC was measured according to Procopet and Pauliuc’s method [65,66]. A 0.2 mL extract aliquot was mixed with 2 mL of 80% methanol and 0.1 mL of 5% AlCl_3_ (prepared in methanol). After 30 min in the dark at room temperature, absorbance was read at 515 nm. Calibration was performed with quercetin solutions (0–10 mg/L), and results expressed as mg quercetin equivalents per kg dry pasta (mg QE/kg).

#### 4.2.7. Pasta Chemical Profile

Moisture, protein, fat, and ash contents were determined according to the International Association for Cereal Science and Technology standard methods, namely: protein (ICC 105/2), moisture (ICC 101/1), fat (ICC 104/1), and ash (ICC 105/1). Carbohydrate content was calculated by difference using Equation (4):

Carbohydrates (%) = 100 − (protein + ash + fat + moisture)(4)

#### 4.2.8. Pasta In Vitro Protein Digestibility

In vitro protein digestibility was determined using a commercial Megazyme^®^ K-PDCAAS enzymatic assay kit (Megazyme, Bray, Ireland), following the manufacturer’s protocol [66]. Pasta samples were milled to a fine powder prior to analysis. Enzymatic digestion was performed sequentially to mimic the gastric and intestinal phases. For the gastric phase, samples were incubated with pepsin solution (1 mg/mL) at 37 °C for 60 min under agitation (300 rpm), followed by intestinal phase incubation using a 1:1 trypsin/chymotrypsin mixture (5 mg/mL) at 37 °C for 4 h (300 rpm). The enzymatic reaction was terminated by heating the samples in a boiling water bath for 10 min in order to deactivate the enzymatic activity. After cooling to room temperature, samples were stored at 4 °C overnight prior to analysis. The concentration of free amino groups released during digestion was quantified using a ninhydrin-based colorimetric assay according to the kit instructions. A calibration curve was constructed using L-glycine (y = 4.3792x + 0.2908, R^2^ = 0.9982).

In vitro protein digestibility (%) was calculated using the following Equation (5):

In Vitro Digestibility (%) = (M × X + B) × 100(5)

The in vitro PDCAAS score was calculated by multiplying the in vitro digestibility by the limiting amino acid ratio (lysine in our case) following Equation (6):

PDCAAS (%) = Digestibility × Ratio × 100(6)

where PDCAAS is the in vitro Protein Digestibility Corrected Amino Acid Score and Ratio is the ratio of the limiting amino acid.

The selected amino acid composition considered for limiting amino acid ratio determination is provided in Table 6. As per PDCAAS calculation, it was performed following the FAO reference amino acid scoring pattern, as recommended within the Megazyme K-PDCAAS assay workflow [67].

#### 4.2.9. ATR–FTIR Analysis

Infrared spectra of RPFC-enriched pasta samples were recorded using a Nicolet iS10 FTIR spectrometer (Thermo Scientific, Karlsruhe, Germany) equipped with a diamond ATR crystal. Measurements were performed in absorbance mode to ensure high accuracy of spectral acquisition.

#### 4.2.10. Pasta Sensory Profile Evaluation

The sensory characteristics of the pasta samples were evaluated by 60 semi-trained panelists using a nine-point hedonic scale (1 = “dislike extremely”; 9 = “like extremely”). The evaluation was conducted at the Sensory Laboratory of the Faculty of Food Engineering, Ștefan cel Mare University of Suceava (Romania). All procedures were carried out in accordance with the ethical principles outlined in the Declaration of Helsinki and were approved by the Ethics Commission of Ștefan cel Mare University of Suceava (Approval No. 252/05.05.2025).

#### 4.2.11. Statistical Analysis

All experimental data were processed using the Statistical Package for the Social Sciences (SPSS, version 16.0; SPSS Inc., Chicago, IL, USA). Results are expressed as mean ± standard deviation. Differences between mean values were evaluated using Fisher’s Least Significant Difference (LSD) test at a 95% confidence level.

## Figures and Tables

**Table 1:** Burger’s model parameters of dough samples enriched with various levels of RPFC.

Sample	Creep Phase				Recovery Phase			
	J_e_ (10^−5^ 1/Pa)	J_max_ (10^−5^ 1/Pa)	λ (s)	η (10^5^ Pa⸱s)	J_r_ (10^−5^ 1/Pa)	J_nr_ (10^−5^ 1/Pa)	J_r_/J_max_ (%)	J_nr_/J_max_(%)
Control	10.91 ± 0.12 ^a^	18.03 ± 0.55 ^a^	91.88 ± 0.21 ^a^	8.42 ± 0.10 ^e^	16.59 ± 0.42 ^a^	1.43 ± 0.14 ^a^	92.04 ± 0.35 ^c^	7.96 ± 0.19 ^d^
RPFC5	4.77 ± 0.09 ^a^	8.26 ± 0.47 ^b^	82.04 ± 0.18 ^b^	17.18 ± 0.14 ^d^	3.34 ± 0.38 ^b^	4.92 ± 0.05 ^c^	40.41 ± 0.20 ^b^	59.59 ± 0.09 ^e^
RPFC10	3.11 ± 0.07 ^b^	5.73 ± 0.39 ^c^	71.23 ± 0.15 ^c^	21.35 ± 0.17 ^c^	3.06 ± 0.31 ^c^	2.67 ± 0.07 ^b^	53.40 ± 0.14 ^a^	46.59 ± 0.07 ^a^
RPFC15	2.69 ± 0.05 ^c^	4.42 ± 0.28 ^d^	67.21 ± 0.09 ^d^	26.64 ± 0.22 ^b^	2.33 ± 0.09 ^d^	2.09 ± 0.03 ^d^	52.71 ± 0.05 ^d^	47.28 ± 0.16 ^b^
RPFC20	1.96 ± 0.04 ^d^	3.94 ± 0.11 ^e^	59.24 ± 0.07 ^e^	29.94 ± 0.31 ^a^	2.16 ± 0.06 ^e^	1.78 ± 0.05 ^e^	54.81 ± 0.07 ^e^	45.19 ± 0.04 ^a^

Data are expressed as mean ± standard deviation (SD) (*n* = 3); different superscript letters in the same column indicate significant difference among samples (*p* < 0.05); J_e_—elastic compliance; J_max_—maximum creep compliance (total compliance at end of creep); λ-retardation time; η-viscosity; J_r_—recovered compliance; J_nr_—non-recovered compliance; J_r_/J_max_—recovery ratio; J_nr_/J_max_—non-recovery ratio; RPFC—rapeseed protein–fiber concentrate; RPFC5, RPFC10, RPFC15, RPFC20—samples with various levels of RPFC addition.

**Table 2:** Thermomechanical behavior of RPFC-enriched dough samples.

Parameters	Control	RPFC5	RPFC10	RPFC15	RPFC20
WA (%)	55.0 ± 0.46 ^a^	69.7 ± 0.76 ^b^	70.8 ± 1.13 ^c^	87.0 ± 0.37 ^d^	90 ± 0.05 ^e^
C1 torque	1.119 ± 0.00 ^a^	1.003 ± 0.10 ^a^	1.210 ± 0.007 ^a^	1.185 ± 0.04 ^a^	1.098 ± 0.01 ^a^
Dough stability (min)	10.20 ± 0.076 ^a^	11.70 ± 1.00 ^ab^	12.10 ± 0.34 ^b^	11.80 ± 0.45 ^b^	12.10 ± 0.15 ^c^
C2 torque (Nm)	0.561 ± 0.01 ^a^	0.606 ± 0.06 ^ab^	0.782 ± 0.02 ^c^	0.688 ± 0.02 ^bc^	0.676 ± 0.01 ^bc^
C3 torque (Nm)	2.173 ± 0.03 ^c^	2.092 ± 0.02 ^c^	2.052 ± 0.02 ^c^	2.009 ± 0.09 ^b^	1.813 ± 0.00 ^a^
C4 torque (Nm)	1.800 ± 0.14 ^c^	1.890 ± 0.02 ^c^	2.058 ± 0.02 ^c^	1.631 ± 0.01 ^b^	1.294 ± 0.01 ^a^
C5 torque (Nm)	3.370 ± 0.19 ^d^	3.000 ± 0.00 ^c^	3.016 ± 0.01 ^c^	2.329 ± 0.04 ^b^	1.755 ± 0.03 ^a^
α slope (Nm/min)	−0.104 ± 0.04 ^a^	−0.072 ± 0.01 ^a^	−0.112 ± 0.00 ^a^	−0.114 ± 0.02 ^a^	−0.080 ± 0.03 ^a^
β slope (Nm/min)	0.410 ± 0.04 ^a^	0.418 ± 0.02 ^a^	0.418 ± 0.00 ^ab^	0.412 ± 0.00 ^ab^	0.358 ± 0.01 ^a^
γ slope (Nm/min)	−0.094 ± 0.04 ^a^	−0.016 ± 0.01 ^a^	−0.062 ± 0.02 ^a^	0.022 ± 0.00 ^a^	−0.016 ± 0.00 ^a^

Data are expressed as mean ± standard deviation (SD) (*n* = 3); different superscript letters in the same column indicate significant difference among samples (*p* < 0.05); RPFC—rapeseed protein–fiber concentrate; RPFC5, RPFC10, RPFC15, RPFC20—samples with various levels of RPFC addition.

**Table 3:** RPFC-enriched pasta’s technological parameters.

Sample	OCT (min)	WA (%)	CL (g)	SI (%)
Control	14.00 ± 0.50 ^c^	168 ± 1.50 ^a^	5.04 ± 0.06 ^a^	1.91 ± 0.03 ^a^
RPFC5	13.30 ± 0.50 ^c^	178 ± 1.11 ^abc^	5.35 ± 0.15 ^a^	2.12 ± 0.02 ^b^
RPFC10	12.00 ± 0.60 ^bc^	163 ± 3.28 ^ab^	8.80 ± 0.70 ^ab^	2.16 ± 0.01 ^b^
RPFC15	11.30 ± 1.04 ^ab^	175 ± 2.51 ^bc^	10.40 ± 0.59 ^bc^	2.16 ± 0.09 ^bc^
RPFC20	10.00 ± 0.00 ^a^	182 ± 1.76 ^c^	5.91 ± 0.04 ^b^	2.31 ± 0.02 ^c^

Data are expressed as mean ± standard deviation (SD) (*n* = 3); Different superscript letters in the same column indicate significant difference among samples (*p* < 0.05). RPFC—rapeseed protein–fiber concentrate; RPFC5, RPFC10, RPFC15, RPFC20—samples with various levels of RPFC addition; OCT—optimal cooking time; WA—water absorption; CL—cooking loss; SI—swelling index.

**Table 4:** DPPH, TPC, and TFC of RPFC-enriched pasta samples.

Sample	DPPH (%)	TPC (mg GAE/kg)	TFC (mg QE/kg)
Control	1.59 ± 0.95 ^b^	358.29 ± 9.18 ^d^	227.46 ± 11.6 ^c^
RPFC5	1.99 ± 0.84 ^b^	260.03 ± 7.93 ^c^	101.03 ± 10.3 ^b^
RPFC10	2.67 ± 0.54 ^a^	247.52 ± 14.29 ^cd^	59.93 ± 12.81 ^bc^
RPFC15	2.38 ± 0.53 ^a^	164.25 ± 7.65 ^b^	150.99 ± 7.58 ^d^
RPFC20	2.51 ± 0.51 ^a^	125.47 ± 3.60 ^a^	164.71 ± 5.43 ^e^

RPFC—rapeseed protein–fiber concentrate; RPFC5, RPFC10, RPFC15, RPFC20—samples with various levels of RPFC addition; data are expressed as mean ± standard deviation (SD) (*n* = 3); different superscript letters in the same column indicate significant difference among samples (*p* < 0.05).

**Table 5:** In vitro protein digestibility and PDCAAS score of RPFC-enriched pasta samples.

Sample	C1 (mM)	C2 (mM)	CN (mM)	In Vitro Digestibility Score	PDCAAS (%)
Control	0.16 ± 0.014 ^a^	0.60 ± 0.127 ^a^	10.40 ± 0.070 ^a^	0.95 ± 0.007 ^a^	56 ± 0.707 ^a^
RPFC5	0.21 ± 0.007 ^ab^	0.85 ± 0.028 ^ab^	11.60 ± 1.598 ^ab^	0.91 ± 0.000 ^a^	73 ± 0.001 ^b^
RPFC10	0.26 ± 0.007 ^abc^	1.06 ± 0.070 ^bc^	12.30 ± 0.883 ^ab^	0.90 ± 0.007 ^a^	83 ± 0.001 ^c^
RPFC15	0.31 ± 0.007 ^bc^	1.33 ± 0.090 ^cd^	13.70 ± 0.325 ^b^	0.91 ± 0.000 ^a^	92 ± 0.010 ^d^
RPFC20	0.41 ± 0.056 ^c^	1.54 ± 0.636 ^d^	15.1 ± 0.325 ^b^	0.92 ± 0.000 ^a^	99 ± 0.707 ^e^

Data are expressed as mean ± standard deviation (SD) (*n* = 3); different superscript letters in the same column indicate significant difference among samples (*p* < 0.05); C1—concentration of primary amines in the diluted sample (mM); C2—primary amine concentration (mM); CN—corrected primary amine concentration (mM); RPFC—rapeseed protein–fiber concentrate; RPFC5, RPFC10, RPFC15, RPFC20—samples with various levels of RPFC addition.

**Table 6:** Selected amino acid profile of RPFC-enriched pasta formulations.

Sample	Proline (%)	Lysine (%)	Histidine (%)	Arginine (%)
Control	1.20	0.36	0.36	0.65
RPFC5	1.38	0.67	0.49	0.97
RPFC10	1.57	0.98	0.62	1.30
RPFC15	1.75	1.3	0.75	1.62
RPFC20	1.94	1.61	0.89	1.94

Control, RPFC5, RPFC10, RPFC15, and RPFC20 refer to pasta samples obtained by partially replacing wheat flour with RPFC at 0%, 5%, 10%, 15%, and 20%, respectively.

**Table 7:** Chemical profile of RPFC-enriched pasta samples.

Sample	Moisture (%)	Ash (%)	Protein (%)	Fat (%)	Carbohydrates (%)
Control	11.80 ± 0.15 ^c^	0.60 ± 0.01 ^a^	10.40 ± 0.20 ^a^	0.45 ± 0.14 ^a^	76.75 ± 0.04 ^d^
RPFC5	11.40 ± 0.16 ^bc^	0.85 ± 0.04 ^b^	11.60 ± 0.06 ^ab^	0.42 ± 0.01 ^a^	75.73 ± 0.77 ^b^
RPFC10	11.10 ± 0.05 ^b^	1.06 ± 0.03 ^c^	13.40 ± 0.19 ^a^	0.46 ± 0.30 ^a^	73.98 ± 0.09 ^b^
RPFC15	10.70 ± 0.15 ^c^	1.33 ± 0.03 ^d^	15.10 ± 0.36 ^ab^	0.45 ± 0.03 ^a^	72.42 ± 0.13 ^b^
RPFC20	10.40 ± 0.03 ^a^	1.54 ± 0.05 ^e^	17.11 ± 0.65 ^b^	0.41 ± 0.01 ^a^	70.54 ± 0.06 ^a^

Data are expressed as mean ± standard deviation (SD) (*n* = 3); Different superscript letters in the same column indicate significant difference among samples (*p* < 0.05). RPFC—rapeseed protein–fiber concentrate; RPFC5, RPFC10, RPFC15, RPFC20—samples with various levels of RPFC addition.

**Table 8:** Formulation of pasta samples with varying levels of RPFC (per 100 g dry matter).

Sample	Wheat Flour (%)	RPFC (%)	Iodized Salt (%)	Water (%)
Control	97	0	3	55
RPFC5	92	5	3	57
RPFC10	87	10	3	60
RPFC15	82	15	3	60
RPFC20	77	20	3	65

Control, RPFC5, RPFC10, RPFC15, and RPFC20 refer to pasta samples obtained by partially replacing wheat flour with RPFC at 0%, 5%, 10%, 15%, and 20%, respectively.

## Data Availability

The original contributions presented in the study are included in the article; further inquiries can be directed to the corresponding author.
